# The effect of the CCR5-delta32 deletion on global gene expression considering immune response and inflammation

**DOI:** 10.1186/1476-9255-8-29

**Published:** 2011-10-26

**Authors:** Gero Hütter, Martin Neumann, Daniel Nowak, Stefan Klein, Harald Klüter, Wolf-K Hofmann

**Affiliations:** 1 Institute of Transfusion Medicine and Immunology, Medical Faculty Mannheim, Heidelberg University; German Red Cross Blood Service Baden-Württemberg - Hessen, Germany; 2 Medical Department III (Hematology, Oncology), Charité Campus Benjamin Franklin, Berlin, Germany; 3 Medical Department III (Hematology, Oncology), University Medical Centre Mannheim, Heidelberg University, Germany

**Keywords:** Chemokine, CCR5-delat32, Graft versus host disease, transplantation

## Abstract

**Background:**

The natural function of the C-C chemokine receptor type 5 (CCR5) is poorly understood. A 32 base pair deletion in the CCR5 gene (CCR5-delta32) located on chromosome 3 results in a non-functional protein. It is supposed that this deletion causes an alteration in T-cell response to inflammation. For example, the presence of the CCR5-delta32 allele in recipients of allografts constitutes as an independent and protective factor associated with a decreased risk of graft-versus-host disease (GVHD) and graft rejection. However, the mechanism of this beneficial effect of the deletion regarding GVHD is unknown. In this survey we searched for a CCR5-delta32 associated regulation of critical genes involved in the immune response and the development of GVHD.

**Methods:**

We examined CD34+ hematopoietic progenitor cells derived from bone marrow samples from 19 healthy volunteers for the CCR5-delta32 deletion with a genomic PCR using primers flanking the site of the deletion.

**Results:**

12 individuals were found to be homozygous for CCR5 WT and 7 carried the CCR5-delta32 deletion heterozygously. Global gene expression analysis led to the identification of 11 differentially regulated genes. Six of them are connected with mechanisms of immune response and control: LRG1, CXCR2, CCRL2, CD6, CD7, WD repeat domain, and CD30L.

**Conclusions:**

Our data indicate that the CCR5-delta32 mutation may be associated with differential gene expression. Some of these genes are critical for immune response, in the case of CD30L probably protective in terms of GVHD.

## Background

The C-C chemokine receptor type 5 (CCR5) belongs to the super family of the seven-transmembrane G-protein coupled receptors (GPCRs) [1]. It interacts with chemokines that mediate the trafficking and function of memory/effector T-lymphocytes, macrophages, and immature dendritic cells towards sites of inflammation [2]. When bound by their chemokine ligands, these receptors can be internalized, impairing the subsequent ability to bind their ligands. Once internalized, these receptors tend to recycle to the cell surface in time. Most chemokines activate more than one receptor subtype and like other chemokine receptors, CCR5 can bind several chemokines [3]. After activation with small ligands, GPCRs are rapidly phosphorylated at serine and threonine residues within the C-tail and the third intracellular loop [4].

CCR5 has gained prominence as a cofactor for HIV-1 entry. Hence, 74 mutations have been described in this gene up to date including the intensively studied 32 base pair deletion (CCR5-delta32) that introduces a premature stop-codon into the CCR5 locus [5-7]. Epidemiologic studies have shown that the mutation occurs most frequently in the Caucasian population with up to 10-20% heterozygous and 1% homozygous carriers, while it can not be found in the Asian, Middle East, African, and the American Indian population [8]. It is hypothesized that the imbalanced distribution of this allele was caused by environmental selective pressure, resulting in positive selection for the delta32 deletion [9]. Individuals lacking CCR5 display no remarkable illness and, no increased susceptibility towards infectious diseases could be observed until Lim et al. figured out a possible role for CCR5 during infection with the West Nile virus (WNV) [10].

Over the last decade, a large number of reports focusing on the role of chemokines in the context of allograft rejection have been made [11]. Furthermore, the first CCR5 inhibitors have been tested concerning their therapeutic significance in terms of transplantation immunology [12,13]. First clinical data will probably be available soon from a trial introducing the CCR5 inhibitor Maraviroc^® ^into allogeneic hematpoietic stem cell transplantation (HSCT) from the Abramson Cancer Center of the University of Pennsylvania (NIH clinical trial number: NCT00948753).

The CCR5 gene is mapped to the short arm of chromosome 3 amongst a group of genes that encode multiple chemokine receptors [14]. CCR5 up-regulation has been proposed by NF-κB, but recently it was suggested that gene regulation is modified by the cAMP/CREP pathway [15,16]. The effect of the CCR5-delta32 deletion on the expression on other genes has been intensively investigated for CXCR4 [17]. The aberrant gene product from CCR5-delta32 builds an intracellular complex with the CXCR4 receptor preventing the expression on the cell surface. Although the mechanism is well described there is a controversy on the question whether this complex is sufficient to suppress CXCR4. Furthermore, it is unknown whether the deletion influences the expression of other genes or forms complexes with a second or third protein.

Apart from the role in HIV infection, the CCR5-delta32 mutation seems to be a modulator regarding immune responses and transplantation immunology. There has also been proposed an association of the mutation with the occurrence of allograft rejection and protection against graft-versus-host disease (GVHD) [18,19].

For HSCT, testing for at least five HLA genes is required before declaring that donor and recipient are HLA-concordant. However, GVHD can occur even though donor and recipient are HLA-concordant as the immune system is still able to recognize other differences in antigenicity and recipients need intensive immunosuppressive medication to prevent the development of GVHD [20,21]. Although there are advances in the treatment of GVHD, this inflammatory immunoreaction is still responsible for 15% of treatment related mortality [22]. Therefore, understanding and manipulating the mechanisms of GVHD is of important scientific and clinical impact.

Chemokines play a crucial role in the pathogenesis of GVHD after allogeneic HSCT. In experimental models, due to the redundancy of receptor ligand interaction, the deficiency or blockage of a single chemokine does not protect the allograft from acute rejection [18]. However, recent studies have demonstrated that the blockade or absence of a single chemokine receptor does prolong allograft survival in a fully HLA mismatched model [23].

However, the molecular basis of the protective effect of CCR5-delta32 is poorly understood. It is still unclear, whether the CCR5-delta32 deletion may have an effect on the expression of genes, which communicate immunological responses or whether the protective effect of the CCR5-delta32 deletion is solely caused by the lack of functional CCR5. One of the most elaborately investigated but also controversially discussed association of the CCR5-delta32 deletion is the putative suppression of the chemokine receptor CXCR4 [17]. With concern to this, there is only data available from an animal model, in which CCR5 has been blocked by specific inhibitors [24,25].

To investigate the molecular basis of the CCR5-delta32 deletion in terms of the biology of immune responses, we performed an array based global analysis of gene expression in CD34+ hematopoietic progenitors from healthy individuals either wild type for CCR5 or heterozygous carriers of the CCR5-delta32 deletion.

Some functional or evolutionary related genes are closely localized in gene clusters. There is a rising count of deletions in other genes described where not only the deletion but also deletion associated down-stream alterations of clustered genes are affected [26,27]. Chemokine and chemokine receptor genes are also known to be clustered in the human genome. Most CC-chemokine receptor genes like CCR5 have been shown to map within 3p21.3, while CXC-chemokine receptor genes were mapped with few exceptions to 2q35 [28]. The clustering of chemokine and chemokine receptor genes suggests a relatively recent and rapid evolution of both gene families by genomic duplications [29]. To detect a potential effect of the deletion on co-regulated or clustered genes, only genes meeting one of two criteria: 1. role in immune response or GVHD or 2. located on chromosome 3, have been selected for further statistical analysis.

## Materials and methods

### Material

Input material for this analysis were immunomagnetically purified CD34+ cells from bone marrow aspiration of 19 healthy volunteers, 9 male 10 female, aged 19-85 (median 25) years. All donors gave written informed consent before investigation. Prior to CD34+ selection, mononuclear cells were isolated by density gradient centrifugation through Ficoll-Hypaque (Biochrom, Berlin, Germany).

### CCR5 genotyping analysis

Genomic DNA was extracted from heparinised peripheral blood monocytes (PBMC) of the donors with the QIAGEN-Blood-Midi-Kit (Qiagen, Germany). Screening of the donors for the CCR5-delta32-allele was performed with a genomic PCR using primers flanking the site of the deletion (forward: 5'-CTCCCAGGAATCATCTTTACC-3', reverse: 5'-TCATTTCGACACCGAAGCAG-3') leading to a PCR fragment of 200 base pairs (bp) for the CCR5-allele and of 168 bp in case of a delta32 deletion. Results were confirmed by allele specific PCR and by direct sequencing using the BigDye^®^-Terminator-1.1.-Cycle-Sequencing-Kit (Applied Biosystems, Germany). Sequences were analyzed using the Vector-NTI-Contig-Express-software (Invitrogen, Germany).

### RNA preparation and array based gene expression analysis

Total RNA was extracted from purified CD34+ haematopoietic progenitor cells using TRIzol (Invitrogen, Karlsruhe, Germany) according to the manufacturer's protocol. The quality of RNA was determined by the 2100 Bioanalyzer system (Agilent Technologies, Waldbronn, Germany) and only samples showing no RNA-degradation were included into the analysis. Oligonucleotide microarrays (HG-U133plus 2.0, Affymetrix Inc., Santa Clara, CA) were hybridized as described previously [30]. Data analysis was performed by the Microarray Suite 5.0 (Affymetrix), and the Genespring software 4.2 (Silicon Genetics, Redwood City, CA). The quality control parameters were in accordance to the MIAME consensus criteria for micro array data with a present call rate of at least 25% [31,32].

### Statistics

All samples were normalized with expression values raised to an arbitrary value of 1. Only expression values which reach a present call rate of 75% have been used. Only genes with a significant (p < 0.05) difference of expression between the CCR5 wild type and the CCR5-delta32 group were eligible for further statistical analysis. Expression analysis of the different groups was performed by using the Mann Whitney test.

## Results

### CCR5 genotyping

From 19 healthy donors, 12 individuals were found to be homozygous for CCR5 WT whereas 7 carried the CCR5-delta32 deletion allele.

### Micro-array analysis

**Figure 1:**
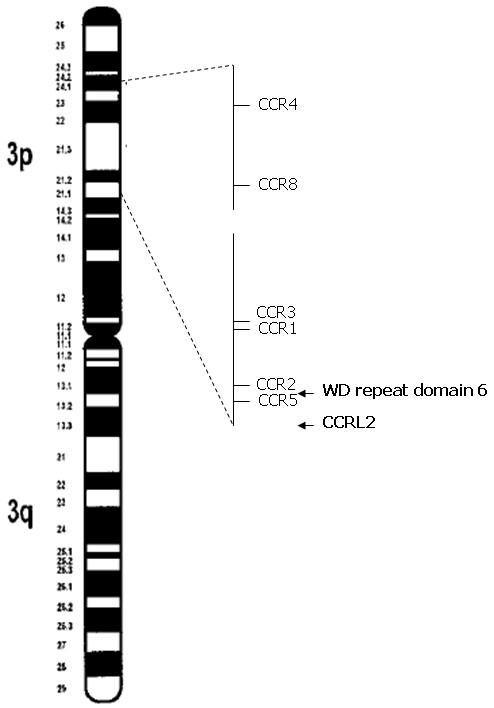
**Mapping the chemokine cluster on chromosome 3**. The differentially expressed genes CCRL2 and WD repeat domain 6 are closely related to the CCR5 gene.

A total of 110 genes were found to be differentially expressed comparing WT and heterozygous carriers of CCR5-delta32. Eleven genes showed a significant higher expression in the wild type group and 99 genes were detected with a higher expression in the CCR5-delta32 group [data not shown]. Further review of gene databases concerning the known or proposed function of these genes regarding immune system and GVHD or location on chromosome 3 revealed 5 genes in the WT group and 6 genes in the CCR5-delta32 group with significantly different expression profiles (Table 1). Of these 11 genes, 5 were located on chromosome 3 and moreover two, CCRL2 and WD repeat domain, in the 3p region closely to the CCR5 gene (Figure 1).

**Table 1:** Gene expression and gene function

Significant higher Expression in CCR5 wild type samples				
	**Gene**	**Location**	**Function**	**Ref**.

1.	LRG1	19p13.3	Protein-protein interaction, signal transduction, and cell adhesion and development. Expression during granulocyte differentiation.	[35]
2.	CXCR2	2q35	Receptor for interleukin 8 and chemokine ligand 1. Mediates neutrophil migration to sites of inflammation.	[44]
3.	HSP70-2	6p21.3	Located in the MHC complex class III region, in a cluster with two closely related genes which encode similar proteins.	[45]
4.	CCRL2	3p21	Encodes a chemokine receptor like protein, most closely related to CCR1.	[46]
5.	RSRC1	3q25.32	Spliceosome assembly and participate in multiple steps of mRNA splicing.	[47]

Significant higher Expression in CCR5-delta32 samples				

1.	CD6	11q13	Involved in T-cell activation.	[48]
2.	CD7	17q25.2-q25.3	Found on thymocytes and mature T cells. Mediates T-cell interactions and also in T-cell/B-cell interaction during early lymphoid development.	[49]
3.	CD30L	9q33	Early CD30 signalling is critical for Treg-mediated acute GVHD protection after major MHC-mismatch HSCT.	[50]
4.	SIAT1	3q27-q28	Catalyzes the transfer of sialic acid from CMP-sialic acid to galactose-containing substrates.	[51]
5.	ATP6V1A	3q13.31	Necessary for protein sorting, zymogen activation, receptor-mediated endocytosis, and synaptic vesicle proton gradient generation.	[52]
6.	WD repeat domain	3p21.31	Interacts with serine/threonine kinase 11, and is implicated in cell growth arrest.	[53]

**Figure 2:**
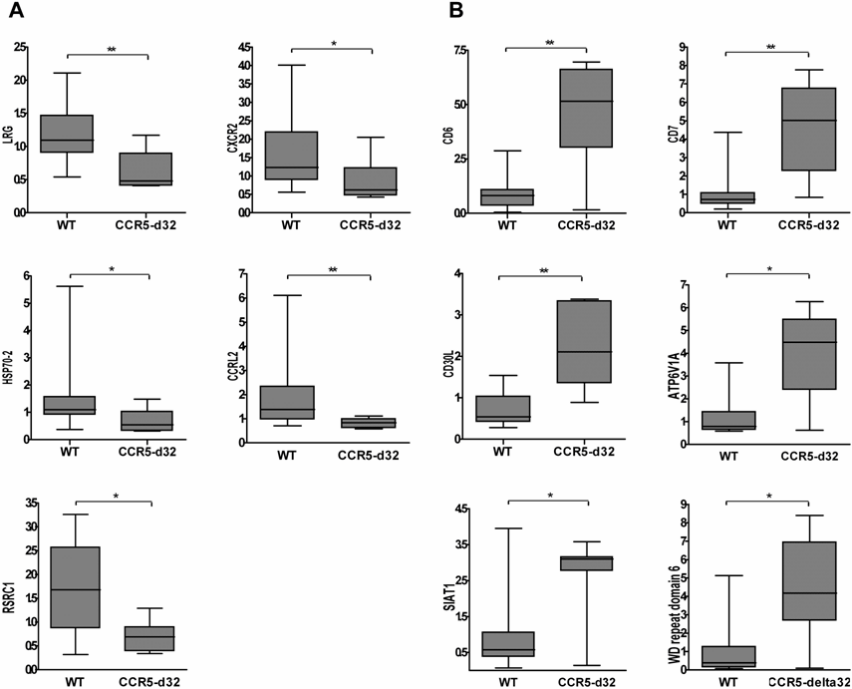
**CCR5 gene array expression analysis**. A. Genes with higher expression in the wild type (WT) group compared to the CCR5-delta32 heterozygous group. B. Genes with higher expression in the CCR5-delta32 heterozygous group compared to the WT group.

The 5 genes with higher expression in the WT group showed a particularly broad variation of expression values whereas in the corresponding group of CCR5-delta32 samples, the variation coefficient was 50% compared to 67% in the wild type group (Figure 2A). In the other group of 6 genes, it was noticeable that only small values of expression were detectable in the WT group compared to those obtained from the CCR5-delta32 samples (Figure 2B)

## Discussion

The protective effect of alteration of CCR5 expression in patients after allogeneic HSCT and recipients of allografts has been proposed in several circumstances (Table 2). There, acute and chronic graft failure in recipients of allogeneic organs was significant reduced in the group of CCR5-delta32 patients. Furthermore, a significant association of the common CCR5 haplotype (H1/H1) and advantage of disease free survival and overall survival in recipients of allogeneic HSCT could have been found. The authors suggested CCR5 genotyping as a new diagnostic and prognostic strategy for therapy optimization [33].

**Table 2:** Transplantation and CCR5 polymorphism

No. of patients	Setting of transplantation	Genotyping Recipients (R)/Donor (D)	Outcome	**Ref**.
Hematopoietic stem cell transplantation				

1370	HSCT (MURD)	(R): CCR5(H1/H1)	DFS ↑, OS ↑	[33]
		(D): CCR5(H1/H1)	DFS ↓	
349	HSCT (MURD & MRD)	(R): CCR5-delta32	GvHD ↓	[19]
		(D): CCR5-delta32	No acute GvHD*	
1273	HSCT (MURD)	(R): nd		[54]
		(D): CCR5-delta32	GvHD ↓**	

Solid organ transplantation				

158	Liver	(R): CCR5-delta32	AR ↓	[55]
		(D): nd		
1227	Kidney	(R): CCR5-delta32	Allograft Survival ↑	[18]
		(D): nd		
163	Kidney	(R): CCR5-59029-A/G	AR ↓	[56]
		(D): nd		
158	Heart	(R): CCR5 No-E	EAR ↓	[57]
		(D): nd		

Summary of clinical trials focusing on the outcome of allogeneic stem cell or tissue transplantation in regard to different CCR5 polymorphism. In most cases, CCR5 genotyping was only performed in recipients. (HSCT = hematopoietic stem cell transplantation, MURD = matched unrelated donor, MRD = matched related donor, nd = not done, DFS = disease free survival, OS = overall survival, GVHD = graft-versus-host disease, AR = acute rejection, EAR = early acute rejection)

* in the case of CCR5-delta32 for both donor and recipient, respectively.

** not significant

The search for host factors and their genetic contribution to immune responses presumes fundamental understanding of pathogenesis. The association of candidate gene polymorphisms in several circumstances has provided new strategies of intervention. In the future, genomic tests will allow performing both, prognostic and predictive sub-typing of patient populations and of HSCT donors, respectively. Patients may benefit from especially selected donors targeted to their specific disease processes and will (probably) therefore have a reduced risk regarding development of life-threatening adverse events like GVHD.

In terms of immune activation, we found two genes (LRG1 and CXCR2) with significant higher expression in the wild type group, which are related to leukocyte differentiation and trafficking. LRG1 works as a secretory type 1 acute-phase protein whose expression is up-regulated by the mediator of acute-phase response [34]. However, its role in the context of GVHD is still to be determined. The LRG1 gene is localized to chromosome 19p13.3, a region to which the genes for several neutrophil granule enzymes also map and that has a proposed role in early neutrophilic granulocyte differentiation [35]. For CXCR2 there are data available on the effect of CXCR2 polymorphism in the context of GVHD. The transcriptional activity of the CXCR2 variant promoter was 2.6-fold higher than that of the wild-type promoter. However, no significant association was observed between CXCR2 polymorphisms and allograft outcomes [36].

Interestingly, three of the six genes overexpressed in the CCR5-delta32 group are important in T-cell (CD6 and CD7) or both T- and B-cell function (CD30L). For example, CD30 and its ligand CD30L may be an important co-stimulatory molecule and marker for the physiological balance between TH1/TH2 immune response associated with allograft rejection [37]. The CD30/CD30L pathway is a potent regulator of CD4+, but not CD8+, T cell-mediated GVHD. Although blocking CD30/CD30L interaction in vivo did not affect alloreactive CD4+ T cell proliferation or apoptosis, a substantial reduction in donor CD4+ T cell migration into the gastrointestinal tract was readily observed with minor effects in other GVHD target organs [38]. However, the role of CD30L and the CD30/CD30L interaction in immune response has still to be determined and recent studies in an CD30^-/- ^animal model or pharmacological blockade of CD30/CD30L interaction are somehow contradicting [39]. Nevertheless, the CD30/CD30L interaction has been found to be critical for the suppressive effect on GVHD of CD4+CD25+ Treg (T regulatory) cells [40]. It has been assumed that Treg cells are one of the key players in reducing GVHD while preserving antitumor activity of memory CD8+ cells after allogeneic HSCT [41].

Here, we could describe two genes, CCRL2 and WD repeat domain 6, flanking the CCR5 gene on chromosome 3, which showed an altered expression profile comparing wild type and CCR5-delta32 heterozygous group. This could be evidence for a cluster co-regulation of these gene caused by the 32 base pair deletion in the CCR5 gene.

The other four altered genes HSP70-2, RSC1, SIAT1, ATP6V1A have no described association with immune responses or GVHD so far. HSP70-2 which is located in the HLA class III complex deciphers a putative role in autoimmune diseases [42,43].

## Conclusions

CCR5-delta32 deletion in hematopoietic stem cells might be associated with differential expression and some of these genes, such as CD30L, may have additional effects in the development of allogeneic immune responses. In terms of a personalized medicine, the long-term objective will probably be to perform additional screening for less favourable or beneficial genetic polymorphism with regard to an optimized donor selection for recipients of an allogeneic HSCT to prevent treatment related complications like GVHD.

## Competing interests

The authors declare that they have no competing interests.

## Authors' contributions

GH designed this survey, performed the CCR5 genotyping and wrote the manuscript. MN analyzed the micro array data, DN performed the micro array. SK, HK, and WKH critically revised the manuscript for important intellectual content. All authors have read and approved the final version of the manuscript.
